# A Case of an Enigmatic Pulmonary Infiltrate

**DOI:** 10.7759/cureus.828

**Published:** 2016-10-13

**Authors:** Uroosa Ibrahim, Amina Saqib, Michel Chalhoub, Jean Paul Atallah

**Affiliations:** 1 Department of Hematology/Oncology, Staten Island University Hospital; 2 Pulmonary/Critical Care, Staten Island University Hospital

**Keywords:** mycobacterium avium complex, splenic lymphoma, lung cancer

## Abstract

The differential diagnosis of a pulmonary mass in an immunosuppressed host with a history of cancer is broad and includes malignant, infectious and inflammatory etiologies. *Mycobacterium avium *complex (MAC) is a rare cause of opportunistic infection in susceptible individuals that can present as either localized or disseminated disease. On radiologic studies, the pulmonary disease can manifest as heterogeneous linear or nodular densities, a mass-like lesion, or thin-walled cavitary lesions. We present the case of pulmonary MAC in a patient with a history of lung cancer requiring lobectomy, and splenic lymphoma being treated with chemotherapy, presenting with extreme fatigue and a fludeoxyglucose (FDG)-avid mass on positron emission tomography–computed tomography (PET-CT). The patient had a CT-guided biopsy of the mass that demonstrated non-caseating granulomas followed by a right middle lobe transbronchial biopsy that upon histologic examination revealed mild acute and chronic inflammation, and necrotizing caseating granulomas. The acid-fast culture of bronchoalveolar lavage showed the growth of acid-fast bacilli that were identified by deoxyribonucleic acid (DNA) probe as *Mycobacterium avium *complex*.* We discuss the typical radiological manifestations of MAC as well as the role of immunosuppression and B cell-depleting therapy from the predisposition to infection.

## Introduction

Immunosuppression predisposes patients to uncommon infections that do not clinically manifest in patients with an intact immune system. The cause of this susceptibility includes cancer and its associated treatment, where in certain cases patients remain at risk of serious infection up to months after treatment. Examples include lung cancer whereby patients can develop localized airway damage and subsequently, a predisposition to infection; chemotherapy and immune therapy that can lead to prolonged leukopenia and inability to contain infections. We present one such case with a history of lung cancer and diffuse large B cell lymphoma developing *Mycobacterium avium *complex (MAC) infection along the postsurgical lobectomy suture site presenting with extreme fatigue and an FDG-avid mass on PET scan. An informed consent was obtained from the study participant.

## Case presentation

**Figure 1:**
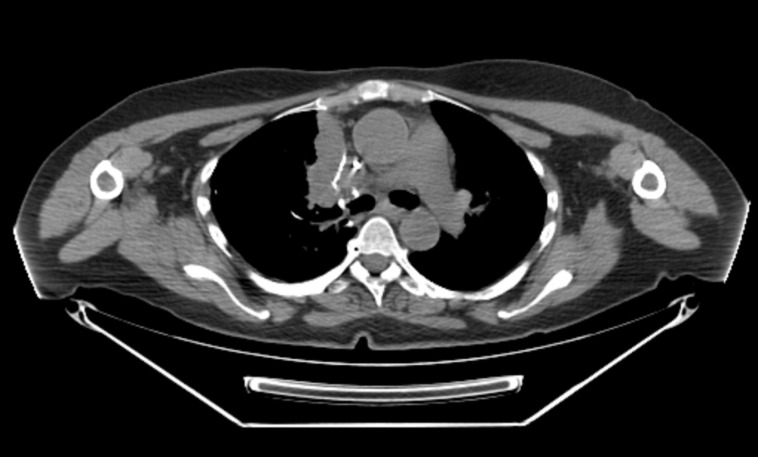
CT scan of the chest in May 2016 A paramediastinal mass, 6.3 x 2.5 cm, is seen.

**Figure 2:**
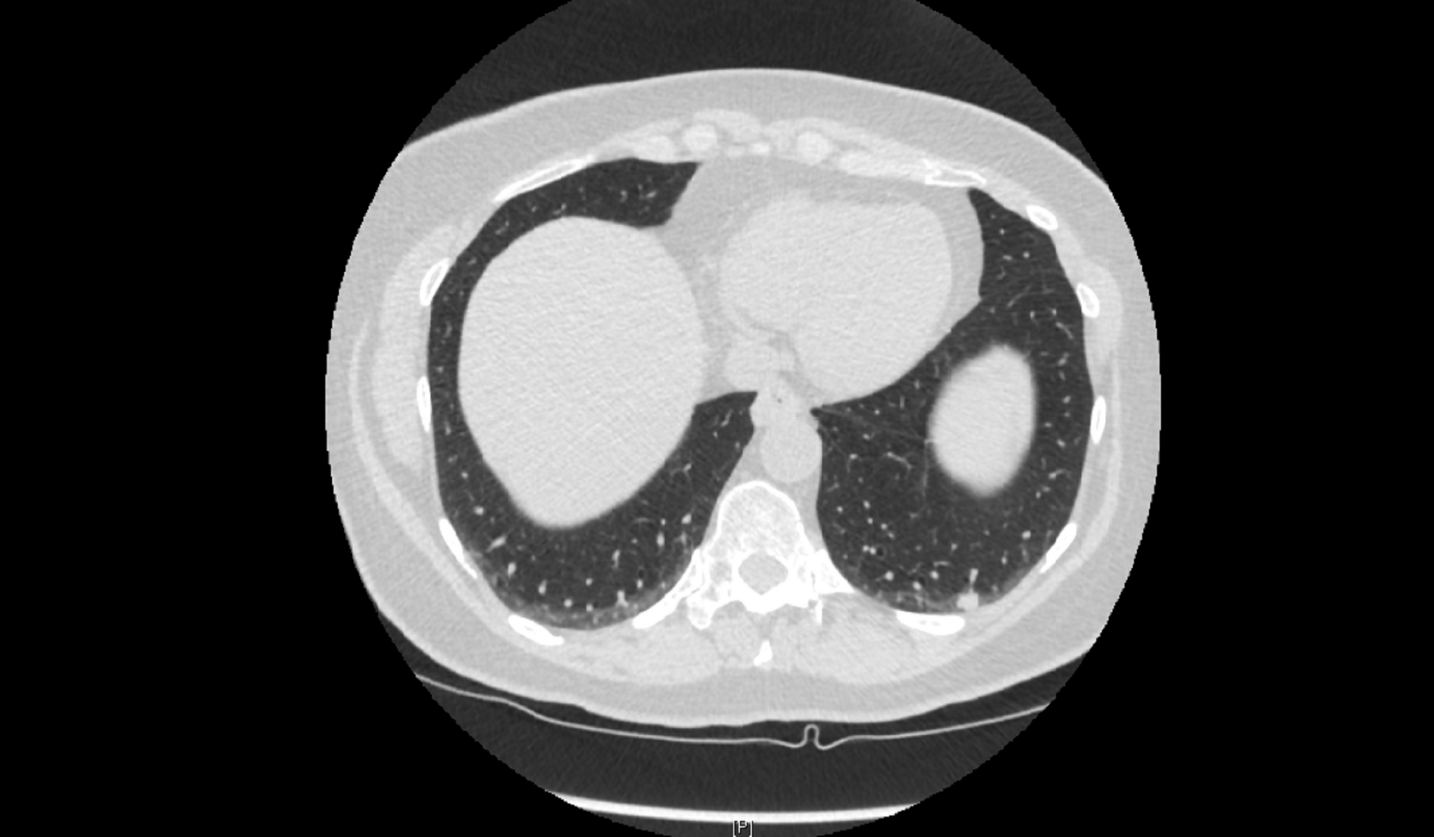
CT scan of the chest in November 2011 New 9 mm solid nodule in the posterior left lower lobe is seen.

A 67-year-old female being followed up for an extensive pulmonary and oncologic history had a PET scan done for follow-up of splenic lymphoma that revealed an FDG avid 6.3 x 2.5 cm paramediastinal mass with an  standardized uptake value (SUV) of 12.4 (Figure 1). The patient was an ex-smoker with a 30-pack-year history of smoking. The patient’s history went back to 2009 when she had a chest-CT scan which showed three nodules, 10 x 4.9 mm, 7.7 x 6.9 mm and 6.8 x 6.1 mm, in the right upper lobe. This was followed by a PET-CT, a month later, which showed mild FDG uptake in all three nodules (maximum SUV of 2.9) with no change in size. The patient continued having surveillance scans until November 2011 when her CT-chest showed a new 9 mm solid nodule in the posterior left lower lobe (Figure 2), with an SUV of 3.0 on a PET-CT that followed.

**Figure 3:**
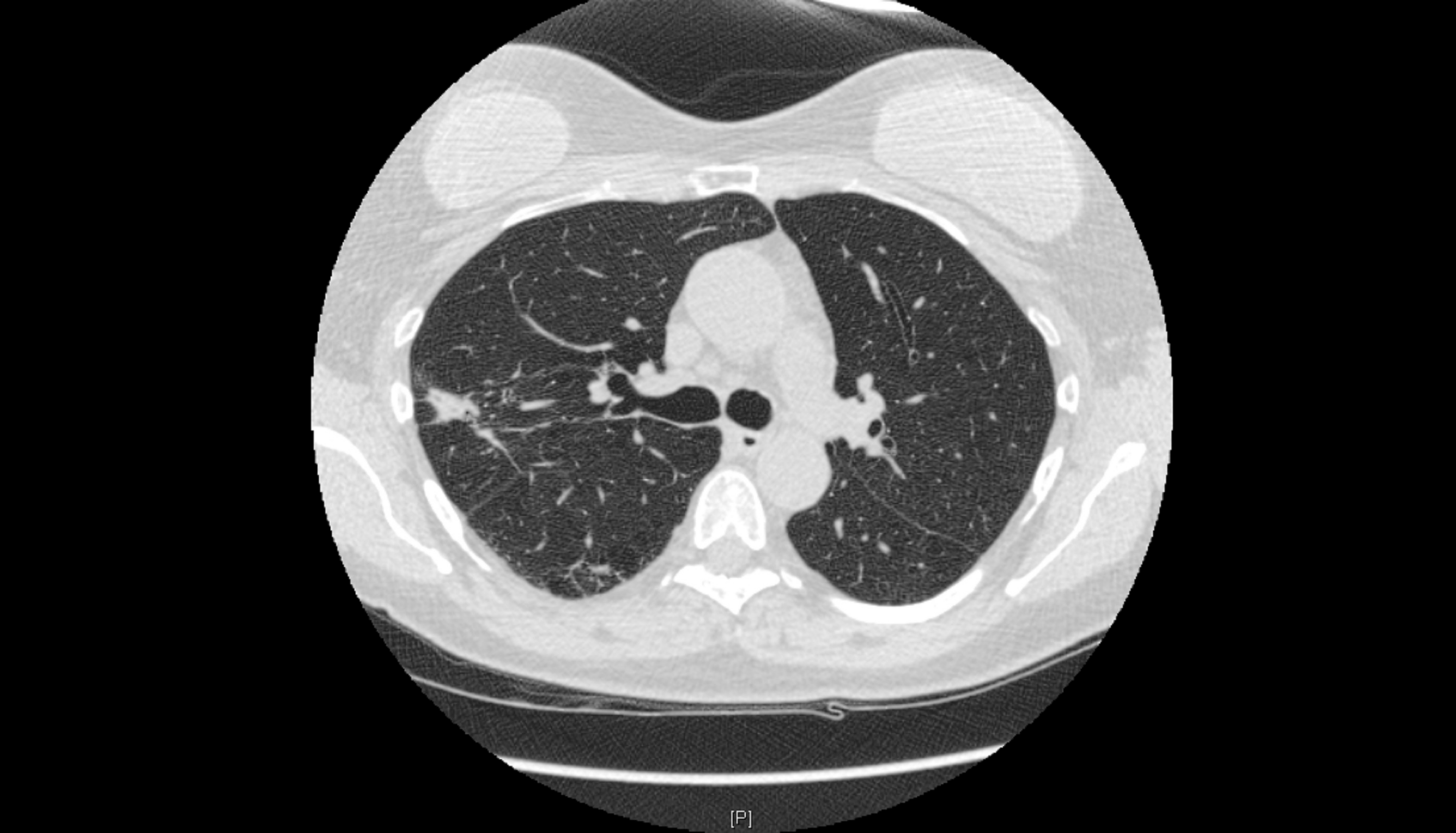
CT scan of the chest in March 2013 A solid right upper lobe 2.0 x 1.0 cm nodule is seen.

**Figure 4:**
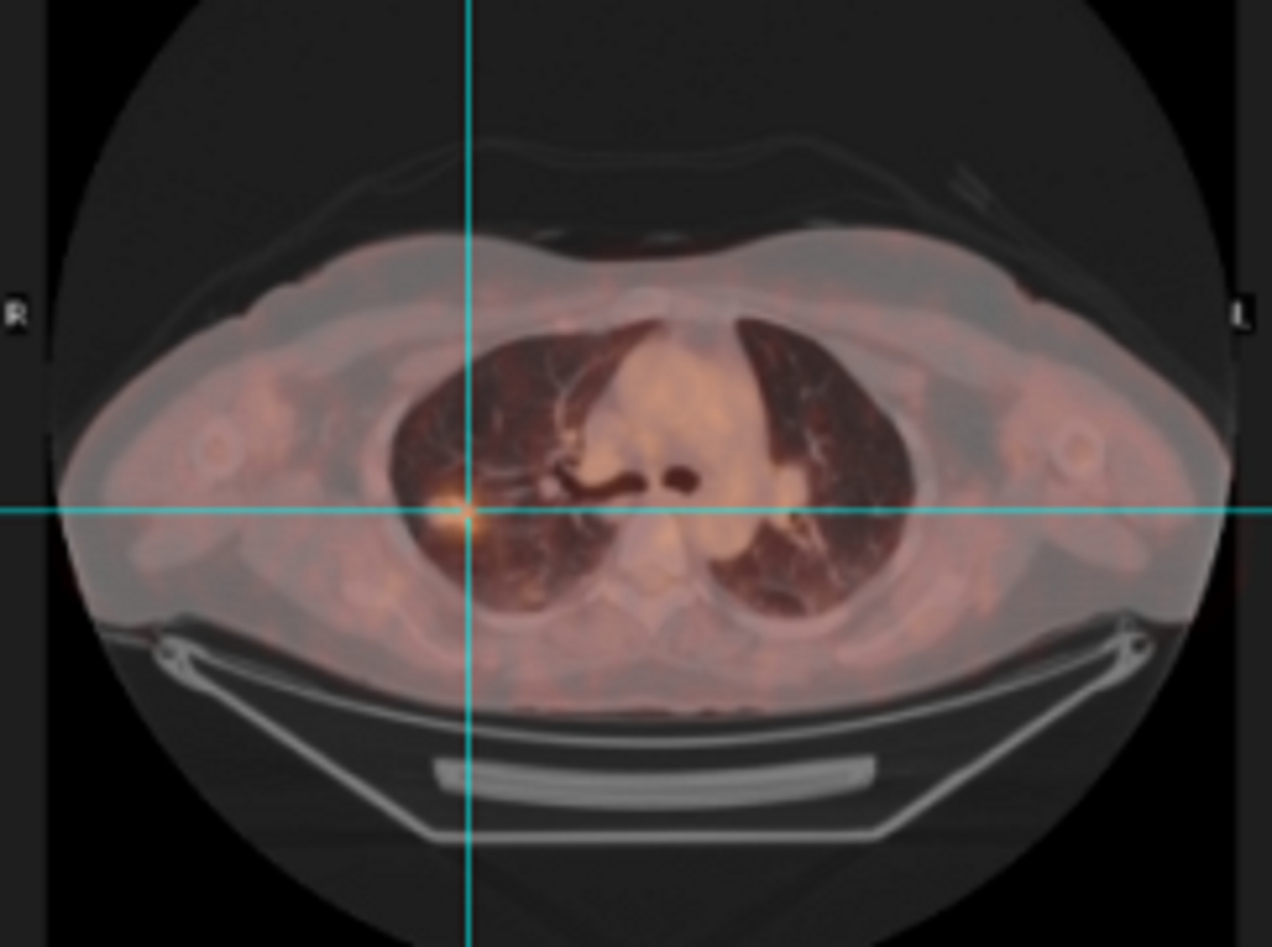
PET/CT scan in March 2013 An FDG-avid right upper lobe nodule with a maximum SUV of 5.2.

In January 2012, the patient underwent left lower lobe wedge resection with mediastinal lymph node sampling. The pathologic examination was consistent with well to moderately differentiated adenocarcinoma with all lymph nodes negative for tumor, hence staged as IA (T1a, N0, M0). She continued periodic follow-up, and a CT-chest scan in March 2013 showed an increase in the size of the right upper lobe nodule to 2.0 x 1.0 cm, solid in appearance (Figure 3). The lesion was FDG-avid on PET scan with a maximum SUV of 5.2 (Figure 4). Following this, in April 2013, the patient underwent right upper lobectomy, with the pathologic examination consistent with well-differentiated adenocarcinoma. As the patient had stage IIb (T3 N0 M0) disease, it was recommended that the patient must receive an adjuvant chemotherapy, which the patient declined.

**Figure 5:**
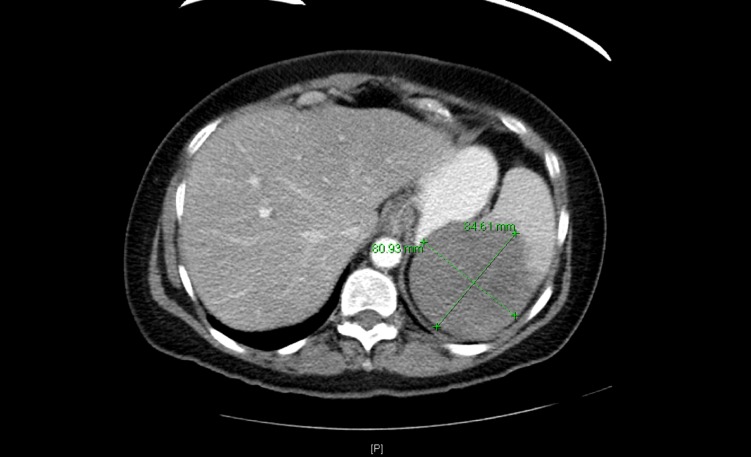
A CT scan of the abdomen in October 2015 A hypodense mass measuring 8.5 x 8.1 x 8.8 cm is seen within the spleen.

**Figure 6:**
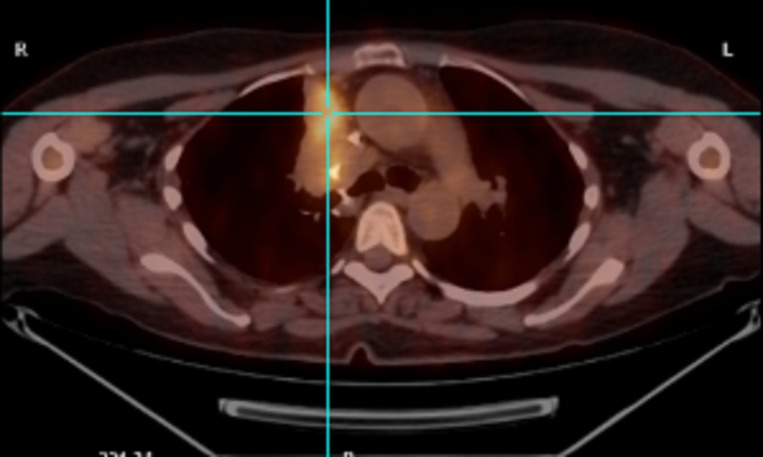
PET/CT scan in May 2016 Pathologic uptake seen within the enlarged right paramediastinal soft tissue mass.

The patient had a repeat imaging in August 2013 and January 2015 that did not show any evidence of disease. In October 2015, the patient presented to the hospital with an acute onset of abdominal pain. A CT scan of the abdomen showed two hypodense masses within the spleen measuring 8.5 x 8.1 x 8.8 cm along the superior aspect and 5.5 x 4.8 x 6.9 cm along the inferior aspect (Figure 5). Several enlarged sub-centimeter lymph nodes were noted within the splenic hilum. A CT scan of the chest done during the same hospitalization showed postsurgical changes with no new nodules. On PET-CT, the splenic masses were FDG-avid with a maximum SUV of 26. An ultrasound-guided biopsy of the spleen was consistent with diffuse large B cell lymphoma with c-myc and bcl-6 positive by FISH. Her lactate dehydrogenase (LDH) was 750 U/L, ESR 65 mm/hr and C-reactive protein (CRP) 4.29 mg/dl. The patient was started on chemotherapy with rituximab, cyclophosphamide, doxorubicin, vincristine and prednisone. After five cycles, the patient had significant fatigue requiring a 10% reduction in the cyclophosphamide dose of her last cycle. A PET-CT scan post-chemotherapy showed resolution of the previously FDG-avid splenic masses. However, pathologic uptake was seen within the enlarged right paramediastinal soft tissue mass along the right upper lobectomy suture line (Figure 6).

**Figure 7:**
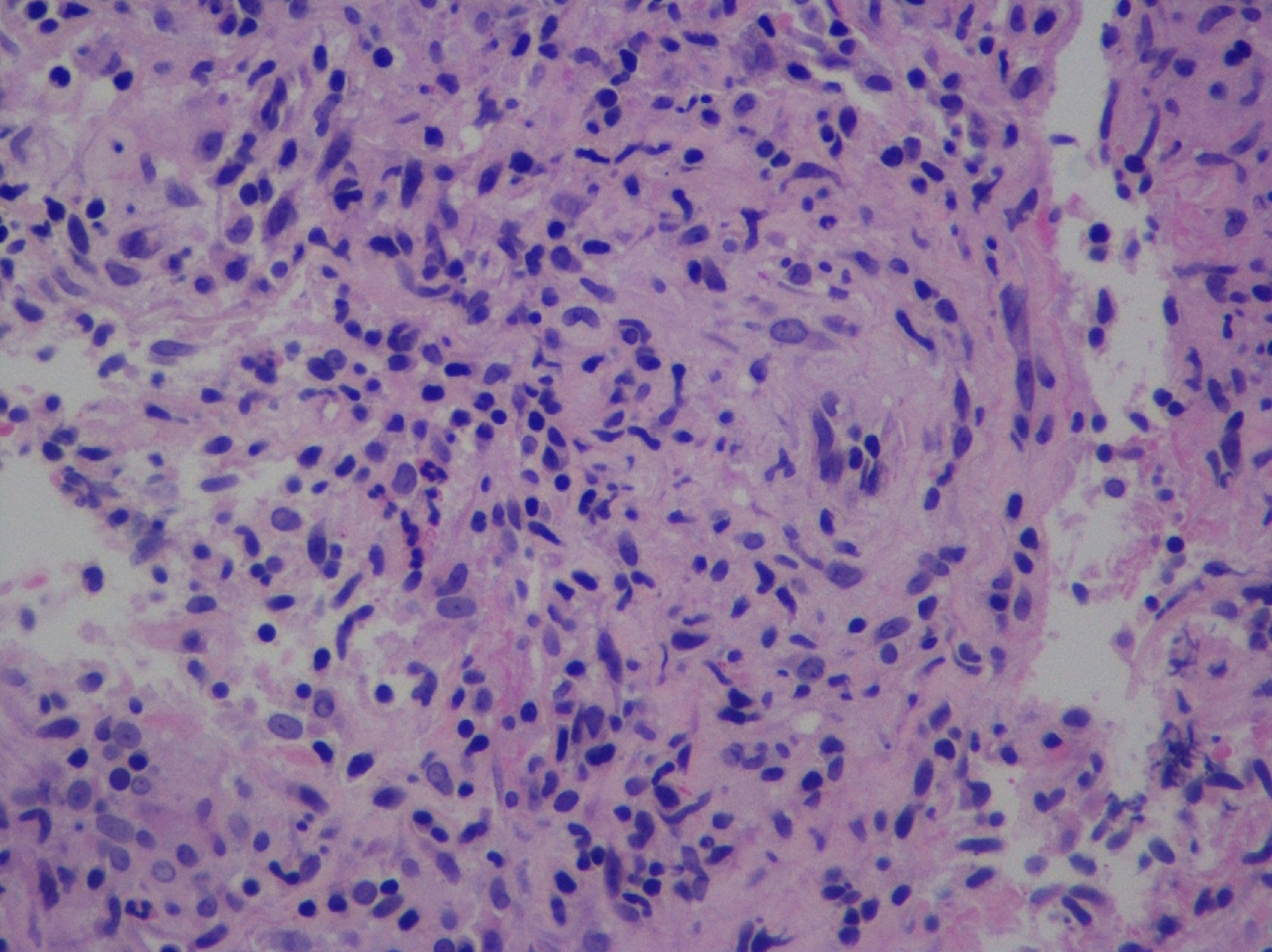
Transbronchial biopsy tissue on histologic exam Necrotizing caseating granulomas seen on a background of mild acute and chronic inflammation.

The mass was concerning for lung cancer recurrence prompting a CT-guided needle biopsy with pathology consistent with non-caseating granulomas, negative for lymphoma or other malignancy and negative for AFB and fungal stains. Following this, the patient underwent a right middle lobe transbronchial biopsy that on histologic exam showed mild acute and chronic inflammation, and necrotizing caseating granulomas (Figure 7). The acid-fast culture of the bronchoalveolar lavage fluid showed growth of acid-fast bacilli (AFB). The patient was started on four-drug anti-tubercular therapy i.e. isoniazid, rifampin, ethambutol, and pyrazinamide. The AFB were identified by DNA probe as *Mycobacterium avium *complex, and the patient was thereafter treated for MAC with clarithromycin 500 mg twice daily, ethambutol 1000 mg once daily and rifampin 600 mg once daily. Two months into diagnosis, the patient remains in remission for her lymphoma and does not have any overt signs or symptoms of infection with the pulmonary lesion stable in size and appearance on follow-up CT scan of the chest.

## Discussion

Cancer and its treatment are associated with an immunosuppressed state that often complicates a patient’s disease course. Several infections are known to cause disease mostly in immunocompromised patients. *Mycobacterium avium *complex (MAC) is one such species which can manifest as disseminated or localized infection. MAC is a slow-growing intracellular pathogen and is part of the group of organisms termed non-tuberculous mycobacteria (NTM) distinguishing them from *Mycobacterium tuberculosis*. With advances in laboratory techniques, as well as increasing prevalence of immunosuppression, over 150 NTM species are now recognized [1].

MAC has mostly been observed in patients with HIV/AIDS, renal transplant recipients, patients on immunosuppressive and chemotherapy and hematopoietic stem cell transplant (HSCT) recipients [2]. Sites of involvement may include lymph nodes, lung, skin, musculoskeletal tissue and indwelling catheters. Patients with lung cancer are at increased risk of pulmonary infection with MAC because of localized airway damage. Patients with hematologic malignancies are at increased risk of disseminated infection because of impaired cellular immunity. NTM prevalence is reported to be as high as 1.2% in hematologic malignancies [3]. However, MAC is identified as the most common pulmonary pathogen, a definite diagnosis of which requires isolation of species from lung biopsy or transbronchonchial biopsy (TBB) specimen, TBB with granulomas, or acid-fast bacilli with a culture of NTM from respiratory secretions [4].

Various radiologic manifestations of pulmonary infection have been described. A classic infection in patients with chronic obstructive pulmonary disease or pulmonary fibrosis presents, on computed tomography, as heterogeneous linear and nodular areas of opacity possibly involving multiple segments [5]. The focal areas of homogeneous opacification resembling a mass-like lesion may also be seen. Another manifestation is a thin-walled cavitation that may facilitate endobronchial spread. Immunocompromised patients may develop hilar or mediastinal adenopathy, cavitation and miliary nodules. Our case is also notable for the FDG tracer uptake as seen on PET scan. PET-CT imaging has gained widespread use not only for oncologic indications but also for inflammatory and infectious diseases. An increased FDG tracer uptake signifies glycolysis in activated white blood cells including macrophages, neutrophils and lymphocytes, as well as in tumor cells [6]. Rare cases of FDG-avid non-tubercular mycobacterial infections mimicking cancer have been reported that were diagnosed as NTM with biopsy specimen showing granulomas, acid-fast bacilli (AFB) positive staining and tissue culture positive for NTM [7].

MAC infection has been described in patients treated with the anti-CD20 antibody rituximab that depletes pro-, pre-, immature and mature B lymphocytes. Lutt, et al. reported the first two cases of NTM (MAC and *M. Kansasii*) infection in patients receiving rituximab for refractory myositis [8]. Peripheral B cells are thought to be important in the host defense against mycobacteria. They have been found in the outer portion of granulomas. In B cell knock-out mice infected with tuberculosis, granulomas were not contained and the mice died [9]. However, it is not yet clear how rituximab therapy can promote disease progression or cause NTM disease. Meanwhile, it is important to have a high index of suspicion for diagnosis of an NTM infection in patients undergoing B cell-depleting therapy especially since some of these patients may already be immunosuppressed from their indication of treatment.

According to the American Thoracic Society (ATS) and Infectious Disease Society of America (IDSA), the treatment of MAC infection is dependent on the disease manifestation. Pulmonary nodular infection should be treated with a combination of oral macrolide, ethambutol, and rifampin. Cavitary, severe or previously treated pulmonary MAC may require intravenous or intramuscular streptomycin or amikacin in addition to the above oral agents. Susceptibility testing is recommended if macrolide-resistant MAC is suspected. Treatment is recommended until culture-negative on therapy for one year [10].

## Conclusions

Our case emphasizes the importance of obtaining a tissue diagnosis in a patient with an FDG-avid abnormality in order to distinguish between a new primary neoplasia, a metastatic lesion, cancer recurrence, or something unexpected and benign such as an uncommon infection as seen in our case. 
